# Perspectives on Transdermal Electroporation

**DOI:** 10.3390/pharmaceutics8010009

**Published:** 2016-03-17

**Authors:** Kevin Ita

**Affiliations:** College of Pharmacy, Touro University, Mare Island-Vallejo, CA 94592, USA; kevin.ita@tu.edu; Tel.: +1-707-638-5994

**Keywords:** electroporation, transdermal, drug delivery, pulse, diffusion

## Abstract

Transdermal drug delivery offers several advantages, including avoidance of erratic absorption, absence of gastric irritation, painlessness, noninvasiveness, as well as improvement in patient compliance. With this mode of drug administration, there is no pre-systemic metabolism and it is possible to increase drug bioavailability and half-life. However, only a few molecules can be delivered across the skin in therapeutic quantities. This is because of the hindrance provided by the stratum corneum. Several techniques have been developed and used over the last few decades for transdermal drug delivery enhancement. These include sonophoresis, iontophoresis, microneedles, and electroporation. Electroporation, which refers to the temporary perturbation of the skin following the application of high voltage electric pulses, has been used to increase transcutaneous flux values by several research groups. In this review, transdermal electroporation is discussed and the use of the technique for percutaneous transport of low and high molecular weight compounds described. This review also examines our current knowledge regarding the mechanisms of electroporation and safety concerns arising from the use of this transdermal drug delivery technique. Safety considerations are especially important because electroporation utilizes high voltage pulses which may have deleterious effects in some cases.

## 1. Introduction

The electrical resistance of the stratum corneum (SC) is between 5 and 25 kV/cm^2^ and the electrical breakdown potential is approximately 75–100 V [1]. This and other peculiarities of the skin makes it challenging for drug molecules to reach systemic circulation. Drug delivery scientists are constantly exploring ways to utilize the advantages of transdermal drug delivery in clinical practice. The advantages include avoidance of first pass effect and gastric irritation. Other advantages of transdermal drug delivery include painlessness and noninvasiveness or in the case of microneedles (MNs) minimal invasiveness. The SC is the major hindrance to transdermal/dermal drug delivery [2]. To overcome this barrier, a battery of different approaches has been used including physical and chemical permeation enhancers. Chemical approaches mostly rely on the use of the so-called accelerants or sorption promoters [3,4,5,6].

Chemical penetration enhancers (CPE) are substances that facilitate transdermal drug delivery by perturbing the SC. It has been postulated that these compounds can enhance transdermal drug delivery by perturbing the SC, increasing partition coefficient or increasing solubility. Bhatia and Singh also studied the influence of ethanol, 5% linolenic acid/ethanol, and 5% limonene/ethanol, as well as iontophoresis on the *in vitro* percutaneous absorption of luteinizing hormone releasing hormone (LHRH) [7]. Solutions of 5% linolenic acid/ethanol or 5% limonene/ethanol significantly enhanced the passive flux of LHRH through human epidermis in comparison to the control [7]. The authors also examined the ultrastructure of human epidermis with transmission electron microscopy (TEM). In addition, iontophoretic flux of LHRH through 5% linolenic acid/EtOH and 5% limonene/EtOH treated epidermis increased significantly in comparison to iontophoretic flux through the control epidermis [7].

Elastic, ultradeformable liposome or transfersome^®^, in the widest sense of the word, is an entity which can pass spontaneously through a barrier and transport material from the application site to the destination site [8]. A transfersome^®^, which has no internal source of energy, achieves the same goal by exploiting the naturally occurring “energy gradients” in the skin. The transepidermal water activity difference is the most obvious and probably the most important such gradient. Transfersomes^®^ are attracted into the body by hydrotaxis [8].

Physical approaches for enhancing transdermal drug delivery include sonophoresis [9,10], iontophoresis [11,12,13], MNs [14,15,16,17] and electroporation [18,19]. Physical techniques are sometimes more effective in comparison with chemical enhancers such as low molecular alcohols or aliphatic esters [20]. Sonophoresis refers to the use of ultrasound for transdermal drug delivery enhancement. Based on the frequency used, this technique can be classified into low-, intermediate- and high-frequency sonophoresis. It has been postulated that when ultrasound is applied to the skin, it has the capacity to increase skin permeability through a variety of mechanisms including acoustic streaming, rectified diffusion, cavitation and cellular-level effect. Increased skin permeability leads to facilitated percutaneous penetration of drugs and biologicals. Iontophoresis refers to the use of mild electric current for transdermal drug delivery [14,15]. During iontophoresis, electromigration and electroosmosis are the predominant mechanisms through which ions are driven across the skin into systemic circulation [14]. Electromigration is the term used to describe the repulsion of positively charged cations by anode and negatively charged anions by cathode. Electron fluxes are transformed into ionic fluxes through electrode reactions, and ionic transport is carried out through the skin to maintain electroneutrality [21].

Nanotechnology is finding increased application in transdermal drug delivery research. Until recently, skin barrier function was hypothesized to be mediated mostly by the SC. Recently, it has been shown that epidermal tight junction (TJ), multiple TJ proteins, including occludin, zonula occludins-1, and in particular claudins, are critical factors for epidermal barrier function [22]. One of the biophysical properties of the microenvironment is substrate topology. Biomimetic microenvironment topology can be engineered by chemical or physical patterning [23]. At the nanoscale level, this process is termed nanotopography. Kam *et al.* recently found that nanotopography loosens tight junctions in simple epithelia and significantly increases transepithelial transport of etanercept [24]. Similar studies involving the skin were carried out by Walsh *et al.* [22]. The skin is a more stratified squamous epithelium [22] and the authors showed that there was significant increase in the delivery of the high molecular weight drug etanercept [22].

Carbon nanotubes (CNTs) are an allotrope of carbon in the form of cylindrical carbon molecules [25]. Strasinger *et al.* demonstrated that application of a small electrical bias (−600 mV) to the CNT membrane on the skin resulted in a 4.7-fold increase in clonidine flux as compared to no bias (0 mV) [26]. The authors postulated that during the positive bias state, the membrane is deactivated and, depending on the therapy, delivers no drug or, in the case of clonidine therapy, operates at its first low steady state level [26]. On the contrary, during negative bias state, the drug is pumped through the CNT membrane at a high steady state level by a combination of electroosmosis and electrophoresis [26].

Transdermal MNs create micron sized pores in the skin to enhance delivery of the drug across the skin [27]. MNs are ideal for patient adherence as they do not stimulate nerves which are associated with pain. MNs improve patient compliance as patient with needle phobia will be more likely to apply the patch because of its painlessness. When MNs are fabricated in arrays on a backing that can be applied to the skin like a bandage, the device is called a MN patch. MNs can be divided into four categories: hollow, solid, coated and polymer. Hollow MNs are like regular hypodermic needles but shorter in length. A liquid formulation of the drug is infused through bores in the MNs. Solid MNs are used to create holes in the skin. Subsequently a patch is then applied. Coated MNs are MNs coated with the drug while polymer MNs are made from polymers that can be dissolving, nondissolving, or hydrogel-forming.

Electroporation refers to the transient perturbation of the skin following the application of high voltage pulses [28]. It is a method used to temporarily create aqueous pores in cell membranes, using electric pulses of high voltage and short duration [1]. It has also successfully been used to enhance skin permeability for molecules with different lipophilicities and sizes including high molecular weight biopharmaceuticals (proteins, peptides, and oligonucleotides). Timolol maleate is lipophilic drug with a log*P* value of 2.1 [29]. Denet *et al.* reported when electrodes were placed at 4 mm from the stratum corneum, the flux of timolol obtained with an electroporation protocol of 10 × 400 V–1 ms (24 µg·cm^2^·h) was about five times higher than the flux obtained with passive diffusion (5 µg·cm^2^·h) [30]. Electroporation can be effective for transdermal delivery of ionic as well as nonionic compounds. Mori *et al.* studied the percutaneous transport of an ionic model compound benzoic acid [31]. The authors connected needle and ring electrodes made of Ag/AgCl to an electrode power source and generated exponentially decaying pulses. The electrical pulse was applied to abdominal hairless rat skin at 150–600 V every minute from 4 to 6 h during the 10-h permeation experiment. Electroporation enhanced transcutaneous flux of benzoic acid [31]. The cumulative amount of the drug which permeated through excised hairless rat skin over 4 h was 196.94 ± 10.80 nmol/cm^2^ without electroporation (control). Transcutaneous flux (one hour electroporation treatment protocol) increased to 439.26 ± 38.1, 517.26 ± 50.0, and 1230.26 ± 128.3 nmol/cm^2^ following the use of needle–needle, ring–needle and plate–plate electrode systems respectively [31]. The cumulative amount of the drug delivered over 4 h withn the 30 min-electroporation protocol was 428.76 ± 99.8, 586.06 ± 210.9, and 1182.26 ± 236.7 nmol/cm^2^ for the needle–needle, ring–needle and plate–plate electrode systems respectively [31]. This study also demonstrated that efficacy can be optimized by the shape of electrodes in addition to the application conditions of electroporation [31]. In another interesting experiment, Mori *et al.* studied the influence of electroporation on percutaneous penetration of a non-ionic compound, mannitol [32]. Transcutaneous flux was measured as a function of different positions of electrodes (cathode in receiver or donor compartments and *vice versa*). The authors observed higher flux values when electroporation (500 V) was used compared to passive penetration. When the anode was in the donor compartment percutaneous flux was 120.1 ± 8.3 µg /cm^2^/h [32]. It was postulated that transdermal drug delivery enhancement was as a result of pore production in the skin membrane [32].

During electroporation, the SC is modified leading to increases in electrophoretic mobility, molecular diffusivity, and electrical conductivity. It has been observed that the flash short high voltage electrical burst in transdermal electroporation can be more effective in enhancing transdermal flux of drugs in comparison with the continuous application of low voltage pulse as in the case of iontophoresis [33]. Electroporation-assisted transdermal transport depends on the shape, amplitude, duration, number of electric pulses, as well as the distance between electrodes. Sugibayashi *et al.* investigated electroporation-mediated transdermal delivery of sodium benzoate [34]. Two silver electrodes were connected to an electrical power source, which generated exponentially decaying pulses. The electrodes were placed on the skin surface with a distance of 0.5 cm between both electrodes. After the 4-h passive permeation experiment, an electrical pulse was applied to the rat skin at 300 V every minute for 10 min. Field intensity generated in the stratum corneum by electroporation was determined by a finite element method. Depending on the position of the electrode, the amounts of benzoate at different skin sites following electroporation were 3-, 2-, and 1.5-fold higher, compared with passive penetration [34].

**Figure 1:**
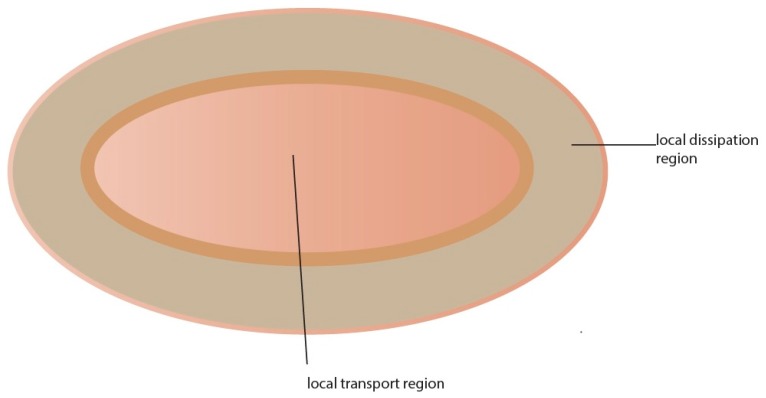
Schematic representation of local transport region (LTR) and local dissipation region (LDR).

Pulse length and amplitude are also influential factors in electroporation when comparing different protocols [35]. It has been shown that molecular and ionic transport across the skin exposed to a number of high voltage pulses is highly localized in sites termed local transport regions (LTRs) [35]. The size of the LTRs (Figure 1) depends on cumulative pulse duration, while pulse amplitude dictates their density. Researchers in the field are in general agreement that there are different responses according to two primary pulsing regimes: short duration-high voltage (HV) pulses result in an altered SC that is perforated with micrometer-sized aqueous pathways while long duration low voltage (LV) pulses result in regions of increased permeability within the SC that are relatively large (up to hundreds of µm) and long lasting [35]. The interaction between the skin and electric pulse in the long pulse regime occurs at much longer timescales (up to hundreds of ms) and is linked to the development of large regions of altered SC (up to hundreds of µm). The large region of affected SC that results from this expansion is termed the LTR. Molecular transport through transiently permeabilized skin by electroporation results from different mechanisms at different times. Enhanced diffusion, during and after pulses, and electrically driven transport during pulses, *i.e.*, electrophoretic movement and very slightly electroosmosis, are the main mechanisms of transport. The contribution of electrophoresis and diffusion are dependent on the physicochemical properties of the molecule [28].

## 2. Mechanisms of Electroporation

Transdermal electroporation, which is also called electropermeabilization, takes place when the skin is exposed to high electric field pulses [36]. Molecules are able to cross the SC when the optimal electroporation parameters are chosen. Temporary electroporation of the skin occurs when the applied external field exceeds the critical transmembrane potential [37]. From the molecular standpoint, the underlying mechanisms that govern structural changes in the skin during electroporation are still not clear. It has however been postulated that water pores form in the skin following application of high voltage pulses. Electroporation of lipid bilayers induces transient increase in transmembrane transport. It has been suggested that when voltage drop across the SC is more than 30 V, the skin experiences a sudden increase (up to four orders of magnitude) in permeability within 10 µs [38]. According to this postulate, modification of SC lipid ultrastructure following application of high voltage pulses occurs due to the interaction between the water dipole and the electric field. [38]. The SC contains approximately 100 bilayer membranes in series and transient increase in permeability usually takes place when voltages of 30–100 V (100–1500 V applied voltages) are used. This is similar to the spectrum of voltages used for cell electroporation, *i.e.*, 0.3–1.0 V per bilayer [28]. Electroporation is a non-thermal process at the level of the cell membrane [39]. When a membrane is subject to electroporation, water is forced into the lipid environment because of the large difference in dielectric constant ($\varepsilon_{water}\approx \;$87,$\;\varepsilon_{lipid}\approx \;$2). As soon as an electropore is formed, the water inside the pore becomes polarized, stabilizing the pore in the process [39].

Most studies, albeit indirectly, propose the existence of “pores” or aqueous pathways created as a result of electroporation. The pores are said to be small (<10 nm), temporary and sparse (0.1% of surface area) [28]. Measurements of the electrical properties of the skin during pulse application have also been proposed as a useful technique for assessing pore evolution [36]. Some investigators have used AC signals to study the passive electrical properties of biological sample before and after electroporation at single frequencies [36]. It has been suggested that short duration-high intensity pulses can lead to an altered SC that is filled with nano- to micrometer-sized aqueous “pores”, and that long duration medium intensity pulses can lead to the creation of regions of increased permeability within the SC that are large (up to hundreds of µm) but with lower density (number of pathways per SC surface area) [38]. The mechanism associated with alteration of the SC lipid structure during the HV pulses is linked to the interaction between the water dipole and the electric field. In one study, it was demonstrated that it is the polarity of water (and not the charge of the lipid head groups) that causes pore formation within the lipid structure [38].

## 3. Electrophoresis

During high voltage pulses, the main driving force for transport of charged molecules is electrophoresis. Evidence for this major contribution of electrophoresis is the drop in the drug transport with reverse electrode polarity opposing electrophoresis [28]. It is being gradually being accepted in the scientific community that the major component in the delivery of charged moderate and large molecules across the skin is electrophoresis [37]. The direct current (DC) portion of the applied electric field provides an electrophoretic force for delivering molecules across the stratum corneum. Field strength, pulse duration, number of pulses , and pulse shape are all parameters of the applied electric field which control both skin permeabilization and drug transport across the skin [37]. The same authors [37] have also observed that a high-field-strength first pulse combined with a longer-duration, lowerfield-strength second pulse can facilitate electroporation efficiency.

## 4. Molecular Diffusion

Molecular transport through electroporation-mediated permeabilized skin is also due to enhanced passive diffusion [28]. Although much higher skin permeability is achieved during the pulse, prolonged permeabilization and thereby transport occur after pulsing, lasting for hours in *in vitro* studies. Evidence for the contribution of enhanced post-pulse diffusion arise from the increased transport seen (i) with reverse electrode polarity, (ii) neutral molecules, and (iii) when the drug is added after application of the pulses [28].

## 5. Electrosmosis

In contrast with iontophoresis, the contribution of electroosmosis during high voltage pulses is low [28]. The short time of current application (few s) limits the role of electroosmosis in drug transport by skin electroporation. Further evidence comes from the similar anodic and cathodic transport of neutral molecules [28].

## 6. Transdermal Electroporation Experiments

**Figure 2:**
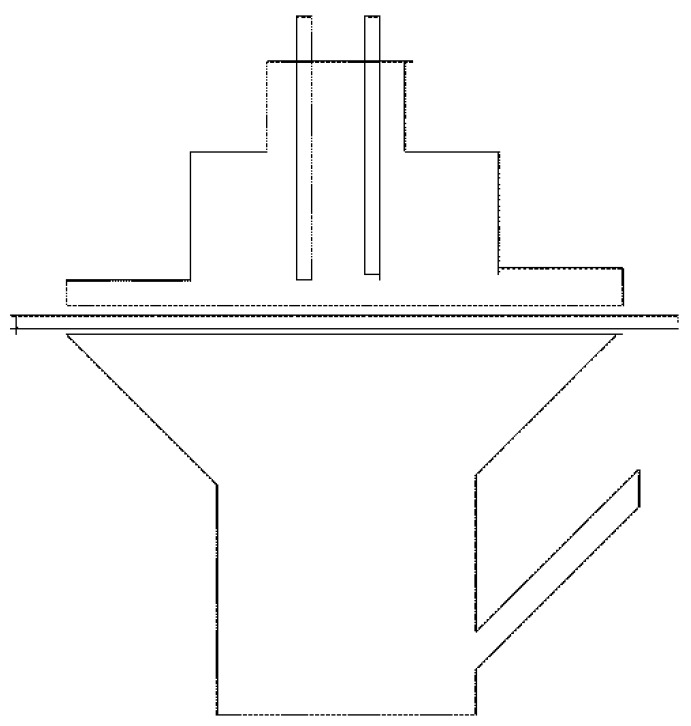
Experimental setup for transdermal electroporation.

Transdermal electroporation experiments typically use Franz diffusion cells or other types of two-compartment diffusion cells (Figure 2). One compartment is the donor compartment where the drug solution is placed and the other contains a receptor solution. Human, porcine, rabbit, guinea pig or mouse skin is then positioned between the two compartments with the SC side facing the donor compartment. Commercial electroporation equipment for performing *in vitro* experiments is usually available as a sealable cylindrical chamber fitted with an electrode plate at each end [33]. A capacitor connected to a power supply system creates an electric charge and the resistors control current and pulse length. The voltage generators can provide voltage in the form of exponential decay or square wave pulses. Square wave pulses have the advantage of pre-adjustment at constant predetermined voltage and pulse length. Silver/silver chloride electrodes are used as they are reversible electrodes [33]. A square wave pulse generator with an array of 7 pin electrodes arranged in a honeycomb configuration has also been used for electroporation [40]. Before starting the experiments, the receptor compartment is typically filled with phosphate buffered saline (PBS, pH 7.4) and maintained at 32 °C (skin temperature) or 37 °C (body temperature) using a water bath. The solution in the receptor compartment is continuously stirred with a magnetic stirrer. Each piece of skin is placed between the donor and receptor compartments and samples taken at different time points (from minutes to two-hourly intervals and up to 72 h) for high-performance liquid chromatographic (HPLC) or liquid chromatographic tandem mass spectrometry (LC–MS) analysis. Next, cumulative amount *versus* time curves plotted and the slopes of the curves used to calculate transcutaneous flux values.

Flux values can be calculated by using the steady-state portion of the cumulative amount *versus* time curves. In some studies, drug concentration is corrected for sampling effects according to the Hayton–Chen equation [41]. This equation has been used by a number of investigators to calculate transdermal flux values [42,43].

(1) $$C_{n}^{1}=C_{n}\left(\frac{V_{T}}{V_{T-\;V_{S}}}\right)\left(\frac{C_{n-1}^{1}}{C_{n-1}}\right)$$

In this equation, $C_{n}^{1}\;$is the corrected concentration and $C_{n}$ represents the measured concentration in the *n*th sample. $V_{T}$ is the total volume of the receiver fluid and $V_{S}$ represents the volume of sample withdrawn from the receiver fluid (1 mL). While $C_{n-1}^{1}$ and $C_{n-1}$ are corrected and measured concentration, respectively in (*n*−1)th sample. 

In transdermal drug delivery experiments, researchers seek to achieve therapeutic drug concentration both *in vitro* and *in vivo*. To illustrate this fact, the target transdermal flux for the antihypertensive agent perindopril can be calculated using an equation that links flux to clearance and steady-state therapeutic concentration [12]. This equation has been used by Gannu *et al.*, as well as Paudel *et al.* [42,44]:

(2) $$J_{ss}=\frac{C_{ss\;\;}\times \;CLt}{A}$$

where $C_{ss}$ is perindopril therapeutic concentration (55 ng/mL), CLt is total body clearance (3.54 L/h) [45,46], A is the surface area of the transdermal patch (10 cm^2^). Using this formula, the target transdermal flux for perindopril will be 19.47 μg·cm^−2^·h^−1^ [12]. An approach towards increasing percutaneous penetration of perindopril using electroporation will seek to achieve this minimum flux.

## 7. Electroporation-Mediated Delivery of Low Molecular Weight Drugs into and across the Skin

Electroporation-mediated transdermal delivery of selected compounds across different skin models is shown in Table 1. Blagus *et al.* used a new *in vivo* real-time monitoring system based on fluorescently labeled molecules for the quantification of transdermal and topical drug delivery [18]. Electroporation of the mouse skin was performed with new non-invasive multi-array electrodes, delivering different amplitudes of electric pulses ranging from 70 to 570 V, between the electrode pin pairs. Patches, soaked with doxorubicin (DOX) or fentanyl (FEN), were applied to the skin before and after electroporation [18]. There was increased transdermal delivery up to the amplitude of 360 V, and then a decrease at higher amplitudes (460 and 570 V) [18]. Topical delivery steadily enhanced with increasing the amplitude of the delivered electric pulses, being even higher than after tape stripping used as a positive control. The non-invasive monitoring of the delivery of DOX, a fluorescent chemotherapeutic drug, qualitatively and quantitatively confirmed the effects of EP at 360 and 570 V pulse amplitudes on topical and transdermal drug delivery. Delivery of FEN at 360 and 570 V pulse amplitudes was similar to effects observed with DOX [18]. The FEN analgesic activity was determined by measuring cornea reflex, pinna reflex, muscle reflex, tail withdrawal latency and FEN plasma levels was less pronounced at the electric pulses with the amplitude of 570 V than at 360 V [18].

Vanbever *et al.* investigated the transdermal delivery of metoprolol permeation through full thickness hairless rat skin *in vitro* following electroporation with an exponentially decaying pulse [47]. Application of electric pulses increased metoprolol permeation as compared to passive diffusion through untreated skin. Raising the number of twin pulses (300 V, 3 ms; followed after 1 s by 100 V, 620 ms) from 1 to 20 increased drug transport. Single pulse (100 V, 620 ms) was as effective as twin pulse application (2200 V, 1100 V or 300 V, 3 ms; followed after 1 s by 100 V, 620 ms. The authors also evaluated the effect of pulse voltage on transcutaneous metoprolol transport [47]. They applied five single pulses (each separated by 1 min) at varying voltages from 24 to 450 V (pulse time 620 ms). A linear correlation between pulse voltage and cumulative metoprolol transported after 4 h suggested that voltage controls the quantity of drug delivered. Then, the effect of pulse time on metoprolol permeation was studied by varying pulse duration of five single 100 V pulses from 80 to 710 ms (each pulse also separated by 1 min [47]. In the same study, it was also demonstrated that pulse time was a contributory factor to enhanced cumulative transport [47].

Sharma *et al.* also studied parameters which influence transdermal delivery of terazosin hydrochloride to hairless rat skin after application of electroporation [48]. The authors found out that the number of pulses, voltage and pulse length (tau) were the most important electroporation parameters. It was proposed that to create a significant transcutaneous flux enhancement, it was necessary to deliver five or more exponentially decaying electroporation pulses, at 88 ± 2.5 V (voltage across the skin), with a decay time constant of 20 ms [48].

Doxepin is a tricyclic antidepressant with analgesic effect in chronic neuropathic pain when used topically. Sammeta *et al.* used electroporation (120 V, 10 ms, 30 pulses at 1 Hz with post pulse waiting period of 20 min) to increase percutaneous penetration of doxepin and doxepin-hydroxypropyl-beta-cyclodextrin (HPCD) complex solution (CDS) across porcine skin [49]. The amount of doxepin retained in the epidermis following electroporation did not differ significantly between pure drug solution and CDS. In studies carried out to ascertain skin accumulation, it was shown that doxepin release attained a plateau within approximately 2.5 days in case of PDS, whereas in case of CDS, doxepin release was prolonged up to five days [49].

Nalbuphine (NA) is a narcotic analgesic agent with a short half-life and a low bioavailability. For these reasons, the drug is usually administered many times a day as an injection. Sung *et al.* investigated the influence of electroporation on transdermal delivery of nalbuphine and its prodrugs [50]. Electroporation was applied using an exponential decay using a pulse generator and platinum electrodes. The electroporation protocol was one pulse per 30 s and applied for 10 min; the pulse voltage was 300 V and pulse duration was 200 ms. The various electrical parameters investigated were pulse voltage, pulse duration and pulse number; the different skin barriers examined were intact hairless mouse skin, SC-stripped skin, delipidized skin as well as furry Wistar rat skin. Application of electroporation significantly enhanced transdermal permeation of NA and its prodrugs. The passive penetration of NA across intact hairless mouse skin was 51.16 ± 5.50 nmol·cm^−2^ while electroporation-assisted flux was 164.13 ± 25.31 nmol·cm^−2^ [50].

Hu *et al.* also investigated the influence of electroporation on percutaneous transport of tetracaine across rat skin [51]. Electroporation (square-wave pulse, voltage 130 V, pulse time 0.4 s, pulse frequency 40 pulses per min) enhanced transdermal diffusion of tetracaine. Percutaneous flux value for the drug at 0.25 h after electroporation (pulse number 400) was 54.6 ± 6 µg·cm^−2^·h^−1^. The corresponding passive flux was 8.29 ± 5 µg·cm^−2^·h^−1^ [51].

It has also been demonstrated in the literature that high voltage electric field pulses can enhance the transdermal delivery of hydrophilic compounds. Timolol is a hydrophilic β-adrenergic blocking agent used in the management of hypertension, arrhythmias and angina pectoris. Denet and Préat studied the examined the feasibility of using electroporation to increase percutaneous penetration of timolol [30]. The authors used square wave pulses and electroporation enhanced the transdermal transport of timolol by one to two orders of magnitude as compared to passive diffusion.

## 8. Electroporation-Assisted Delivery of Macromolecules

Zorec *et al.* studied the influence of the order of different square wave electric pulses on transdermal electroporation. Most electroporation protocols use the same repetitive, mostly exponentially decaying pulses but this study compared different combinations of square wave short high voltage (HV) and longer low voltage (LV) electroporation pulses [18]. *In vitro* experimental results showed that longer LV pulses significantly increased subsequent passive transport of calcein through dermatomed pig skin, while short HV pulses alone resulted in negligible calcein passive transdermal transport. Surprisingly, when the long LV pulses were preceded by short duration HV pulses, the total calcein transported was reduced significantly [18].

Petchsangsai *et al.* studied the influence of electroporation (EP), sonophoresis (SN) and MNs on percutaneous transport of the hydrophilic macromolecular compound fluorescein isothiocyanate-dextran (FD-4; molecular weight (MW) 4.4 kDa) across porcine skin [53]. The authors used 99 pulses of 50, 100, 200, and 300 V with a blunted-MN array serving as an electrode. The pulse interval had a duration of 100 ms. Transdermal flux values following the application of 99 pulses (200 and 300V) were 0.151 ± 0.047 and 0.216 ± 0.085 μg·cm^−2^·h^−1^ respectively. According to the authors, FD-4 skin permeation was undetectable at voltages below 200 V. The authors demonstrated that the total cumulative amount of FD-4 that permeated porcine skin using three combined techniques (MN–EP–SN) was greater than the amount observed using a single method or two combinations (MN–EP, MN–SN, SN–EP). When the three techniques were combined, transdermal flux was 16.58 ± 2.35 μg·cm^−2^·h^−1^.

Sen *et al.* also studied the effect of 1,2-dimyristoyl-3-phosphatidylserine (DMPS) and electroporation on the percutaneous transport of insulin [1]. The epidermis was pulsed for 10 min and the enhancing effect of DMPS on the transport of insulin was examined by measuring the transport in the presence and the absence of DMPS (2 mg/mL). Electroporation was carried out using a pulse generator delivering a single or multiple unipolar square pulses of 100–105 V. The authors found out that when electroporation was carried out in the presence of DMPS, there was greater than 20-fold enhancement of insulin transport. Furthermore, while in the presence of the phospholipid, almost all the transported insulin was detected in the receiver compartment; in the absence of added lipids, only about half the insulin transported was in the receiver compartment and an almost equal amount of insulin remained in the epidermis.

Heparin is a negatively charged anticoagulant (molecular weight 5000–30,000 Da) that is used for the management of thrombosis. Prausnitz *et al.* evaluated the transport of heparin across the SC of cadaver skin after high-voltage pulsing electroporation [52]. Millisecond pulses of 150–350 V given 12 times per min over a period of 1 h increased heparin flux rates of 100–500 µg·cm^−2^·h^−1^.

Electroporation is also widely used in experimental settings for gene transfer into and through the skin. This is usually a two-step process involving skin permeabilization followed by electrophoresis [54]. Several investigators have achieved skin permeabilization by using high voltage (HV) pulses. These pulses are short (about 100 of microseconds) and intense (about 1000 volts per centimeter). After a certain period of time, electrophoresis is carried by the application of low voltage (LV) pulses. These are long (about several hundred of milliseconds) and less intense (about 100 volts per centimeter) pulses [54]. Topical and/or transdermal electroporation may also be clinically relevant for the management of skin disorders, cutaneous cancers, vaccinations and systemic metabolic diseases [55]. Usually, cutaneous gene electrotransfer (GET) is carried out following intradermal DNA injection. Guo *et al.* described plasmid DNA delivery with a multielectrode array (MEA) in a hairless guinea pig model. The authors observed significant increase(up to 4 logs) in gene expression with intradermal DNA administration followed by topical non-invasive skin gene electrotransfer [55]. It is also important to emphasize that in this study, gene expression was observed exclusively in the epidermis [55].

Calvet *et al.* studied the influence of different electrotransfer parameters after intradermal administration of a DNA [54]. The authors investigated a CD8 response-monitoring DNA vaccination model by using the INVAC-1 plasmid which encodes a modified form of the human telomerase reverse transcriptase gene(hTERT) [54]. C57BL/6J mice were injected intradermally with either pCMV-luc (encoding luciferase) or INVAC-1 (encoding a modified form of hTERT) plasmids, followed or not by the application of electric pulses (1 HV pulse of 1000 V/cm and of 100 µs followed 1000 ms later by 1 LV pulse of 140 V/cm and of 400 ms) [54]. The authors measured luciferase expression 48 h after EGT of pCMV-luc and the frequency of the interferon γ (IFNγ) + hTERT-specific CD8 T-cells 14 days after EGT of INVAC-1. There were statistically significant increases in both the luciferase expression and the frequency of IFNγ + hTERT-specific CD8 T-cells following dermal electroporation [54].

There is widespread investigation of the skin as a target for DNA vaccination in a clinical setting due to the immunocompetent nature of the dermis, accessibility of the target and the ease of monitoring [56]. Electroporation in the skin has the benefit of being minimally invasive and generally well tolerated [56]. Electroporation parameters such as electrical field intensity, pulse length, pulse width, and plasmid formulation have great influence on the efficiency of DNA delivery to the skin [56]. Antiangiogenic metargidin peptide (AMEP) is a novel anticancer agent. The antiproliferative and antiangiogenic effects are due to binding to αvβ3 and α5β1 integrins [57]. Spanggaard *et al.* carried out a first-in-man phase I study to investigate safety and tolerability of intratumoral plasmid AMEP electrotransfer into cutaneous metastatic melanoma [57].

Significant increase in the number of new biotechnology macromolecules has not been matched with effective delivery technologies. Electroporation continues to be an important approach in the delivery of genes and other biologically active macromolecules.

## 9. Combination of Electroporation with Other Transdermal Drug Delivery Techniques

Frequently, electroporation is combined with other transdermal/dermal drug delivery techniques to increase transcutaneous flux. Zan *et al.* used surfactants to increase transdermal delivery of a lipophilic drug piroxicam [58]. The drug is frequently “entrapped” in the skin due to its lipophilicity. The authors investigated two surfactants, Tween 80 and sodium dodecyl sulfate(SDS) [58]. It was reported that the use of surfactants led to a 30–50-fold increase in electroporation-mediated percutaneous flux of piroxicam. The authors observed that SDS was more effective than Tween 80 in transdermal delivery enhancement [58]. Tokumoto and coworkers also studied the combined influence of iontophoresis and electroporation on percutaneous transport of insulin [59]. The authors found out that electroporation (150 or 300 V, 10 ms, and 10 pulses) led to a high plasma level of insulin and that the simultaneous use of electroporation and iontophoresis resulted in an additional increase in insulin flux. The flux enhancement resulting from the combination of iontophoresis and electroporation was more compared to the use of electroporation alone [59]. The authors also changed the pH of the system from 7 to 10 and this led to more increase in transdermal drug delivery rate [59].

A combination of electroporation and iontophoresis was used by Fang *et al.* for the transdermal delivery of 5 fluorouracil (5-FU) [60]. Electroporation was performed using an exponential decay pulse generator with platinum electrodes. The electroporation protocol consisted of 1 pulse per 30 s, applied for 10 min. The pulse voltage was 300 V, and pulse length was 200 ms [60]. Twenty 300-V, 200-ms pulses significantly increased 5-FU permeation from compared to passive diffusion. Application of iontophoresis significantly facilitated the transdermal transport of 5-FU with fluxes of 0–31.41 µg/cm^2^/h. The synergistic combination of the two techniques resulted in a higher permeation of 5-FU than either technique alone [60]. Electroporation treatment exerted a disruptive influence on the SC [60].

Tokumoto *et al.* investigated the influence of electroporation and iontophoresis on the percutaneous transport of insulin [59]. Passive diffusion and iontophoresis alone (0.4 mA/cm^2^) resulted in almost no skin permeation of insulin at pH 7 while electroporation (150 or 300 V, 10 ms, and 10 pulses) led to a high plasma level of insulin [59]. The combined use of electroporation and iontophoresis led to a further increase in the plasma level of insulin compared with that measured after electroporation alone [59].

Yan *et al.* investigated the influence of electroporation and MNs on the transdermal delivery of fluorescein isothiocyanate (FITC)-dextran (FD-4: average molecular weight, 4.3 kDa) [61]. MNs were arranged to puncture the skin barrier, the SC, and electrodes were used for electroporation. *In vitro* skin transdermal experiments showed that electroporation in combination with MNs had a higher skin penetration-enhancing effect for FD-4 than MNs alone or conventional electroporation alone [61].

The above-mentioned reports indicate that in certain scenarios, the combination of electroporation with other transdermal drug delivery techniques may lead to percutaneous transport enhancement. Several variables are involved and synergistic flux enhancement can only be proved by experiments. Iontophoresis, MNs and sonophoresis in combination with electroporation seem to be particularly attractive.

## 10. Challenges

Over the past few decades, several researchers have carried out studies aimed at using electroporation to enhance percutaneous penetration of drugs. Even though some measure of success has been attained, there are still challenges. Most transdermal electroporation experiments carried out use either an approximation of different repetitive high voltage plus low voltage protocols (repetitive exponentially decaying pulse protocols) or true square wave repetitive pulses of either high voltage or low voltage configuration [35]. These pulses are of different amplitudes, durations and numbers and therefore interpretation of results must take these differences into consideration.

Another major area of concern is the safety of transdermal electroporation. These experiments involve the use of high voltage pulses and so there have been concerns regarding short-term and long-term safety of transdermal electroporation. Various histological, visual, electrical and microscopic techniques have been employed by a number of researchers to demonstrate the safety of electroporation. Denet *et al.* report that alterations of the skin structure and electrical properties following the use of high voltage pulses are mild and reversible but muscle contractions are sometimes induced [28]. In addition, the same authors also observe that that there are structural changes which persist within the LTR, due to combined thermal and effects. Other observed effects include pain, an increase in skin hydration, disorganization of the SC lipid bilayers, transient impairment of the barrier function as well as a transient increase in the blood flow [28]. Additionally, increased current/charge, pulse rate and pulse length all increase levels of sensation. Because sensation decreases dramatically for pulses shorter than about 1 ms, short pulses, at high-voltage might provide significant increase in transdermal transport without sensation or pain. However, decreasing pulse length strongly decreases transdermal transport. Another approach may involve using an appropriate electrode configuration for sensation reduction or elimination [62].

Skin irritation is another concern in transdermal electroporation. Medi and Singh used the Draize visual scoring system to evaluate erythema and edema following DNA vaccine delivery through electroporation in rabbits. The authors used the Draize system to grade erythema and edema: very slight erythema barely perceptible #1; well defined erythema #2; moderate to severe erythema #3; severe erythema, beet redness to slight eschar formation, injuries in depth #4; and edema: very slight edema, barely perceptible #1; slight edema, edges of area well defined by definite raising #2; moderate edema, area raised approximately 1 mm #3; severe edema, raised more than 1 mm, and extending beyond area of exposure #4 [63]. No erythema or edema was observed with the 100 V electroporation pulses. However, there were edema and erythema with 200 and 300 V electroporation pulses that disappeared by seven days following electroporation [63]. The authors concluded that the mild skin irritation caused by electroporation pulses was reversible [63].

As investigators gain more mechanistic insight into the electroporation process, there is hope that attempts will be made to carry out concomitant toxicological studies to ensure that patient safety is not compromised.

## 11. Conclusions

The SC is a resilient layer of the skin that hinders the ingress of substances into the human body. Because of the elegant structure of the skin, drugs and vaccines cannot easily enter into the body in therapeutic quantities. Among the approaches used to facilitate transdermal drug delivery, electroporation has attracted considerable interest. In this review, the process of electroporation has been discussed and considerable attention focused on the mechanism of transdermal electroporation. Reports of the use of this technique for transdermal delivery of small as well as large molecular weight compounds have also been highlighted. Some researchers have combined electroporation with other percutaneous drug delivery enhancement techniques such as iontophoresis, sonophoresis or microneedles. In somes cases, flux values increased due to the synergies created by combined technniques. It is also interesting to know the types of structural changes that occur in the skin when two or three techniques are combined. Another area of interest is the relationship between electroporation and skin irritation. Since high voltage pulses are used, it is important to ensure that there are no harmful effect on the skin. In this regard, several researchers have used histological, visual, electrical and microscopic techniques to demonstrate the safety of electroporation. Although, there are still challenges, there are reasons to be optimistic that researchers will gain more mechanistic insight into electroporation thereby offering possibilities for eventual clinical use.

## Figures and Tables

**Table 1:** Electroporation-mediated transdermal delivery of selected compounds across different skin models.

Year and Reference	Electroporation Method	Molecular Species	Skin Model
2013, [18]	70 to 570 V electric pulses	doxorubicin, fentanyl, FITC-labelled dextran	mouse skin
1994, [47]	100–300 V	metoprolol	hairless rat skin
2000, [48]	88 ± 25 V	terazosin hydrochloride	hairless rats
2010, [49]	120 V	doxepin	porcine skin
2003, [50]	300 V	nalbuphine	rat skin
2000, [51]	130 V	tetracaine	rat skin
2013, [18]	45 and 500 V	calcein	porcine skin
1995, [52]	150–350 V	heparin	human skin
2003, [31]	150–600 V	benzoic acid	hairless rat skin
2003, [30]	400 V	timolol	human skin
2003, [32]	500 V	mannitol	hairless rat
